# Associations between Dietary Patterns and Bile Acids—Results from a Cross-Sectional Study in Vegans and Omnivores

**DOI:** 10.3390/nu12010047

**Published:** 2019-12-23

**Authors:** Iris Trefflich, Hanns-Ulrich Marschall, Romina di Giuseppe, Marcus Ståhlman, Andreas Michalsen, Alfonso Lampen, Klaus Abraham, Cornelia Weikert

**Affiliations:** 1 Department of Food Safety, German Federal Institute for Risk Assessment, 10589 Berlin, Germany; Alfonso.Lampen@bfr.bund.de (A.L.); klaus.abraham@bfr.bund.de (K.A.); cornelia.weikert@bfr.bund.de (C.W.); 2 Institute for Social Medicine, Epidemiology and Health Economics, Charité-University Medicine, 10589 Berlin, Germany; andreas.michalsen@charite.de; 3 Department of Molecular and Clinical Medicine/Wallenberg Laboratory, University of Gothenburg, 405 30 Gothenburg, Sweden; hanns-ulrich.marschall@gu.se (H.-U.M.); Marcus.Stahlman@wlab.gu.se (M.S.); 4 Institute for Epidemiology, University Kiel, 24105 Kiel, Germany; romina.digiuseppe@epi.uni-kiel.de

**Keywords:** vegan diet, fecal and serum bile acids, dietary pattern, reduced rank regression

## Abstract

Bile acids play an active role in fat metabolism and, in high-fat diets, elevated concentrations of fecal bile acids may be related to an increased risk of colorectal cancer. This study investigated concentrations of fecal and serum bile acids in 36 vegans and 36 omnivores. The reduced rank regression was used to identify dietary patterns associated with fecal bile acids. Dietary patterns were derived with secondary and conjugated fecal bile acids as response variables and 53 food groups as predictors. Vegans had higher fiber (*p* < 0.01) and lower fat (*p* = 0.0024) intake than omnivores. In serum, primary and glycine-conjugated bile acids were higher in vegans than in omnivores (*p* ≤ 0.01). All fecal bile acids were significantly lower in vegans compared to omnivores (*p* < 0.01). Processed meat, fried potatoes, fish, margarine, and coffee contributed most positively, whereas muesli most negatively to a dietary pattern that was directly associated with all fecal bile acids. According to the pattern, fat intake was positively and fiber intake was inversely correlated with bile acids. The findings contribute to the evidence that, in particular, animal products and fat may play a part in higher levels of fecal bile acids.

## 1. Introduction

An increasing trend toward plant-based diets and, in particular, vegan diets was observed in developed countries over the last few years [1,2]. A vegan diet is defined as a diet without consumption of any animal products and is supposed to be rich in fiber and low in fat [3]. Due to this dietary composition, vegans and vegetarians tend to have a lower body mass index (BMI), which is considered beneficial to positive health effects against the onset of obesity or type 2-diabetes [4,5]. Two comprehensive meta-analyses demonstrated that a vegan diet is associated with lower prevalence of cardio-metabolic risk factors [6] and a lower risk of total cancer [7]. Colorectal cancer (CRC) is one of the most common cancer types, and incidence rates are increasing in Western countries [8]. CRC is strongly associated with meat intake, whereas fiber intake shows an inverse relationship [9]. Due to its absorptive and viscous properties, fiber interacts with cholesterol and, thus, with bile acid metabolism [10]. This association between diet and fecal bile acid concentrations was observed in several studies [11,12,13,14].

One step in the complex synthesis of bile acids from cholesterol is the formation of 7-alpha-hydroxy-4-cholesten-3-one (C4) in the liver. C4 is a valid serum biomarker for the synthesis of primary bile acids cholic acid (CA) and chenodeoxycholic acid (CDCA) [15,16]. Primary bile acids are conjugated in the liver with glycine or taurine to increase solubility before excretion into bile; the conjugation ratio of glycine–taurine is about 3:1 in humans [17]. Primary bile acids are released postprandially [18], and bile acid concentrations in plasma increased and remained elevated for a longer period after a high-fat/high-energy intake than after carbohydrate intake [19]. Bile acids build micelles, which contribute to the absorption of intestinal cholesterol, triglycerides, and fatty acids [20]. Bacteria of the intestinal microbiota are involved in dehydroxylation at the C-7 atom of CA and CDCA into the secondary bile acids deoxycholic acid (DCA) and lithocholic acid (LCA). Gut bacteria are also involved in epimerization of ursodeoxycholic acid (UDCA) from primary or secondary bile acids [18].

Approximately 95% of bile acids are reabsorbed from the intestine, transported back to the liver via the portal vein, and excreted into bile, to start the enterohepatic circulation once again. The amount of bile acids excreted with feces is equal to the synthesized amount of 0.2–0.6 g/day in adults [21].

Bile acids can stimulate oxidative stress and DNA damage due to their hydrophobicity [22], and they induce the apoptosis resistance of epithelial colon cells [23], which could be related to both inflammatory bowel diseases and colorectal cancer etiology [17,21,23,24,25,26]. Furthermore, bile acids are also signaling molecules. They are principally involved in metabolic regulation and energy expenditure [27], and they are linked to lipid and glucose metabolism via the farnesoid X receptor (FXR), a regulator of the enterohepatic circulation of bile acids [28].

Nutrients are not consumed separately, and diets consist of combinations of different food groups and nutrients. Therefore, dietary pattern analyses provide a better understanding of nutrient interaction [29]. One method identifying dietary patterns is reduced rank regression (RRR), an established method to derive dietary patterns, which was applied in nutritional epidemiology [30,31]. RRR establishes linear combinations of foods or food items (used as predictor variables) with a maximum of a possible variation in disease-related nutrients or biomarkers (used as response variables) [32,33]. RRR is yet to be used within the context of any fecal biomarkers.

However, to date, the effect of a vegan diet on fecal bile acid levels was only investigated in small short-term (a few days or weeks) intervention studies [34,35], but not in a population adhering to a vegan diet for a longer period—at least one year. In addition, there are no data available on serum bile acids in vegans.

The aim of this study was twofold. Firstly, we aimed to compare serum and fecal bile acid concentrations between vegans and omnivores. Secondly, we aimed to identify dietary patterns explaining the variation in fecal bile acid concentrations to identify combinations of food groups contributing to bile acid levels in stools.

## 2. Materials and Methods

Between January and July 2017, 36 vegan and 36 omnivorous persons were recruited for the Risk and Benefits of a Vegan Diet study (RBVD) at the Federal Institute for Risk Assessment, Berlin (BfR) (see study flow chart, Figure S1, Supplementary Materials). Inclusion criteria were ages between 30 and 60 years and a body mass index < 30 kg/m^2^. Each type of diet was to have been followed for at least one year. Exclusion criteria were acute infection, serious diseases, pregnancy or breastfeeding, and taking of proton pump inhibitors or glucocorticoids. Furthermore, the consumption of at least three portions of meat per week or two portions of meat and two portions of processed meat per week was defined as an omnivorous diet, whereas a vegan diet was defined by no consumption of any animal food products. All participants visited the study center twice. On the first visit, the participants were comprehensively informed about the study details and signed the informed consent. On the second visit, lifestyle and basic characteristics were recorded using questionnaires, and a fasting blood sample of 60 mL was taken. Blood lipids, and parameters of the hemogram were analyzed in 30 mL of the sample in a certificated laboratory on the same day. The remaining half was stored at −80 °C for further analysis for serum bile acids, among other elements. The study was approved by the Ethics Committee of the Charité University Medical Center Berlin (No. EA4/121/16).

### 2.1. Collection of Fecal Samples

The participants received a collection device (Fecotainer^®^, AT Medical BV, Enschede, the Netherlands) to collect an entire stool sample at home on the morning of the second visit to the study center. The time of bowel movement, beginning of storage, and time of delivery to the study center were documented by the participants on a form, as well as the frequency of bowel movements and intake of antibiotics within the last two months. To avoid microbial changes, samples were no older than four hours when processed at the study center. Stool samples were weighed and homogenized for 15 min using a stomacher, partitioned into different aliquots, and stored at −80 °C for further analysis.

### 2.2. Analysis of Bile Acids in Stool and Serum

Determination of bile acids in serum and stool was performed in a randomized and blinded manner at the University of Gothenburg, Department of Molecular and Clinical Medicine/Wallenberg Laboratory, Sweden.

For serum, 50 µL of sample was extracted with 500 µL of methanol containing deuterated internal standards (d4-TCA, d4-GCA, d4-GCDCA, d4-GUDCA, d4-GLCA, d4-UDCA, d4-CDCA, d4-LCA d4-C4; 50 nM of each). After 10 min of vortex and 10 min of centrifugation at 20,000× *g*, the supernatant was evaporated under a stream of nitrogen and reconstituted in 200 µL of methanol–water (1:1).

For feces, 30–80 mg of the sample (wet weight) was extracted in 500 µL of methanol containing 2.5 µM of the internal standards stated above. The extraction was made in 2-mL polypropylene tubes filled with six zirconium oxide beads (3 mm). Extensive homogenization and extraction took place for 10 min at 25 Hz using a TissueLyzer II instrument (Retsch GmbH, Haan, Germany). After centrifugation at 20,000× *g*, the samples were diluted 1:100 in methanol–water [1:1].

The samples were injected (5 µL) and bile acids were separated on a C18 column (1.7 µm, 2.1 × 100 mm; Kinetex, Phenomenex, Torrance, CA, USA) using water with 7.5 mM ammonium acetate and 0.019% formic acid (mobile phase A) and acetonitrile with 0.1% formic acid (mobile phase B). The chromatographic separation started with 1-min isocratic separation at 20% B. The B-phase was then increased to 35% over 4 min. During the next 10 min, the B-phase was increased to 100%. The B-phase was held at 100% for 3.5 min before returning to 20%. The total runtime was 20 min. Bile acids were detected using multiple reaction monitoring (MRM) in negative mode (C4 in positive mode) using a QTRAP 5500 mass spectrometer (Sciex, Concord, Canada), and quantification was carried out using external standard curves.

Non-labeled bile acids were attained from Sigma-Aldrich (Stockholm, Sweden), and the deuterated acids were from CDN Isotopes (Quebec, Canada) or Toronto Research Chemicals (North York, Canada) (d4-TCA).

### 2.3. Dietary Assessment

On the first visit, the participants received a detailed explanation of how to record foods and beverages from the study center, and they were provided with three-day weighed food records and digital kitchen scales to record their diet for two weekdays and one weekend day. All 72 participants fully completed their food records when they returned for the second visit to the study center. The data of the food records were captured in EAT software, version 3.5.5 (University of Paderborn, Paderborn, Germany), where each food item was assigned to the German national food code (Bundeslebensmittelschlüssel Version 3.02, BLS). For food items without available food codes, new codes were generated on the basis of ingredients lists on the packaging or were requested from the producers. Ingredients of cooked dishes were converted from recipes into effective quantities of consumed portions. To estimate the weight of cooked food items, yield and retention factors were taken into account [36]. Yield and retention factors of single food items were summarized into superordinate food groups for simplification (Table S1, Supplementary Materials). After data entry, as well as quality and plausibility checks, the food data were merged with the BLS to assign macro- and micronutrients. All food data were averaged to achieve daily food intakes.

After assigning the food items to the BLS code, they were categorized into 49 food groups according to food groups used in European Prospective Investigation into Cancer and Nutrition (EPIC) Potsdam study [37]. New food groups with items typical for a vegan diet, namely, plant-based milk alternatives, meat alternatives, savory vegetable spreads, and soft drinks, were defined and added to the original food groups; thus, consequently, 53 food groups were available (Table S2, Supplementary Materials). Food groups, with vegan and non-vegan food items such as egg found in pasta, cookies, and sweets, were captured separately for each diet.

### 2.4. Assessment of Lifestyle Characteristics

The body weight, height, and waist circumference of the study participants were measured by trained study personnel. Levels of education, lifestyle characteristics such as smoking behavior, alcohol consumption, and physical activity, and medical history were acquired using computer-assisted questionnaires. Physical activity was defined as the sum of average number of hours spent cycling, doing sports, and gardening during the summer and winter per week.

### 2.5. Statistics

Characteristics of the study population, status of nutrients, food groups, and biochemical continuous variables are presented as means and standard deviation for normally distributed variables or as medians and interquartile range (IQR) for variables which are not normally distributed. Categorical variables are presented as percentages. For categorical variables, chi-square or Fisher’s exact test were used, and, for continuous variables, the Mann–Whitney U test or *t*-test (for normally distributed variables) were applied.

The method of reduced rank regression (RRR) is applied in nutritional epidemiology [32] to derive dietary patterns and to identify the linear combination of predictor variables which explain the largest proportion of variation in response variables [38]. In this analysis, the standardized intake of 53 food groups out of the three-day weighed food records was used as predictors, and log-transformed secondary bile acids DCA, LCA, and UDCA, as well as the sum of primary and secondary conjugated bile acids with glycine or taurine, were as responses. The secondary bile acids were selected due to their association with an increased risk of inflammatory bowel diseases and CRC [17,21,23,24,25,26], and conjugated bile acids were chosen as response variables due to their close relationship to diet [17]. The number of derived patterns is equal to the number of response variables; thus, five patterns were identified. To guarantee that the observed variation of fecal markers mirrored the different profiles of vegans and omnivores, the RRR patterns were derived within the pooled data of vegans and omnivores and not by splitting the data by diet [39]. Trends of biomarker concentrations and macronutrient intakes across pattern scores were calculated in logistic models by categorizing the score into tertiles. To calculate the dietary pattern, each food group with a factor loading >0.2 was identified and taken into account.

All statistical analyses were conducted using SAS (version 9.3, SAS Institute Inc., Cary, NC, USA).

## 3. Results

### 3.1. Study Population

In total, 36 vegans and 36 omnivores were included in the RBVD study. The main characteristics of the study population are described in Table 1. No significant differences in body weight, BMI, education level, physical activity, and smoking status were observed between the vegan and omnivorous study group.

### 3.2. Intake of Macro-Nutrients and Food Groups Derived from Three-Day-Weighing Records

Total energy intake did not differ significantly between vegans (*p* = 0.49). Vegans had a significantly lower intake of fat (87.6 mg/day, IQR 64.1–116.2), compared to omnivores (fat 104.1 mg/day, IQR 87.8–143.3) (*p* = 0.024). Fiber intake was significantly higher in vegans (45.6 g/day, IQR 33.7–58.2) than in omnivores (23.7 g/day, IQR 18.6–29.9) (*p* < 0.0001). Median intakes of all food groups are shown in Table S3 (Supplementary Materials).

### 3.3. Concentrations of Bile Acids in Feces and Serum

**Figure 1:**
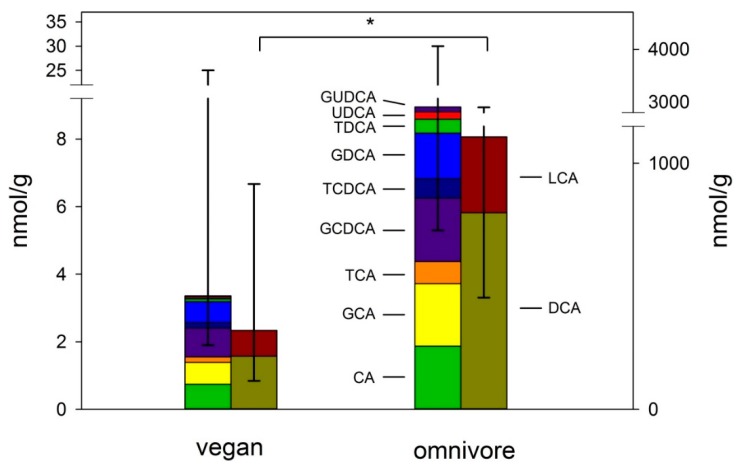
Fecal bile acid concentrations in vegans and omnivores of the Risk and Benefits of a Vegan Diet study (RBVD) study (nmol/g). Data are presented as medians and interquartile range (IQR) for total fecal bile acids. Mann–Whitney U test was used for bile acids (* *p* ≤ 0.001). Values for secondary bile acids are presented on right *y*-axis, while primary and conjugated bile acids are presented on left y-axis. CA= Cholic acid, GCA = glycine-conjugated CA, TCA = taurine-conjugated CA, GCDCA = glycine-conjugated Chenodeoxycholic acid, TCDCA = taurine-conjugated CDCA, DCA = Deoxycholic acid, GDCA = glycine-conjugated DCA, TDCA = taurine-conjugated DCA, UDCA = Ursodeoxycholic acid, GUDCA = glycine-conjugated UDCA, LCA = Lithocholic acid, DCA = Deoxycholic acid.

The fecal bile acid profiles showed significant differences between a vegan and omnivorous diet (Figure 1). Vegans had significantly lower levels of total bile acids (564 nmol/g, IQR 195–1261) than omnivores (1667 nmol/g, IQR 804–5092) (*p* < 0.01). In particular, all secondary and both glycine- and taurine-conjugated bile acids were lower in vegans than in omnivores (*p* < 0.01). Levels of primary CA tended to be higher in the omnivorous group (1.87 nmol/g, IQR 0.57–6.82) than in the vegan group (0.74 nmol/g, IQR 0.18–9.43) (*p* = 0.18). Moreover, there was no difference in CDCA between vegans (0.00 IQR 0.00–2.93) and omnivores (0.00, IQR = 0.00–0.40) (*p* = 0.77).

**Figure 2:**
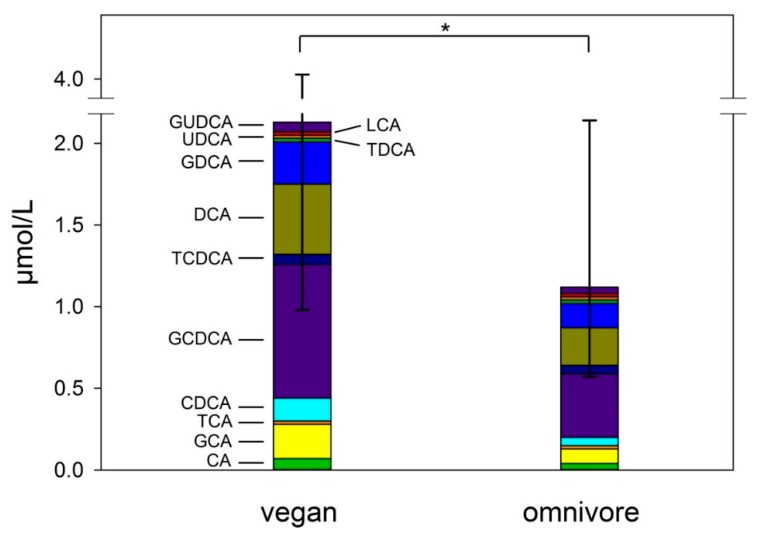
Serum bile acids concentrations in vegans and omnivores of the RBVD study (µmol/L). Data are presented as medians and IQR for total serum bile acids. Mann–Whitney U test was used for bile acids (* *p* ≤ 0.001). CA = Cholic acid, GCA = glycine-conjugated CA, TCA = taurine-conjugated CA, CDCA = Chenodeoxycholic acid, GCDCA= glycine-conjugated CDCA, TCDCA = taurine-conjugated CDCA, DCA = Deoxycholic acid, GDCA = glycine-conjugated DCA, TDCA = taurine-conjugated DCA, UDCA = Ursodeoxycholic acid, GUDCA = glycine-conjugated UDCA, LCA = Lithocholic acid.

Vegan participants had significantly higher total bile acid serum concentrations than omnivores (*p* = 0.001) (Figure 2). Primary bile acids CA (*p* = 0.002) and CDCA (*p* = 0.01) were higher in vegans compared to the omnivorous group. Vegans had higher levels of total glycine-conjugated bile acids (1.32 µmol/L, IQR 0.81–2.53) than omnivores (0.79 µmol/L, 0.47–1.04) (*p* = 0.001). The secondary bile acids DCA, LCA, and UDCA did not differ significantly between vegans and omnivores. C4 levels in serum did not differ between vegans (35.62, IQR 23.19–50.91) and omnivores (30.13 nmol/L, IQR 16.21–47.41) (*p* = 0.36).

### 3.4. Dietary Pattern Explaining Variance in Fecal Bile Acids

**Figure 3:**
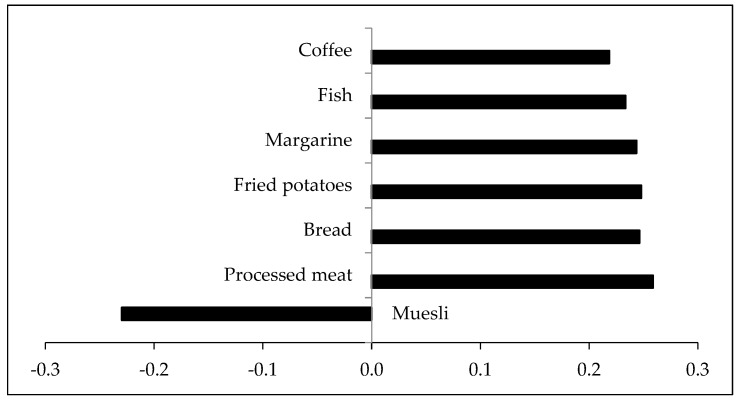
Factor loadings of all 53 food groups in first dietary pattern score. Factor loadings are correlations between food groups and the dietary pattern score. Food groups with factor loadings >0.2 were chosen for the dietary pattern.

The first reduced rank regression-derived dietary pattern explained 47.4% of the variance in bile acids, mostly driven by the explained variance in glycine-conjugated bile acids (69.1%) and taurine-conjugated bile acids (54.8%). This pattern consisted of positive loadings for coffee, fish, margarine, fried potatoes, bread, and processed meat, and a negative loading for muesli (Figure 3). Factor loadings of all food groups are presented in Table S4 (Supplementary Materials).

The concentrations of DCA, LCA, and conjugated bile acids used as response variables were significantly higher across the tertiles of the dietary pattern score (Table 2), whereas the proportion of vegans was lower across the score. The first tertile included 75% vegans, the second tertile included 50% vegans, and the third tertile included 25% vegans.

Fiber intake decreased across the tertiles of the first dietary pattern (*p* = 0.01). Intakes of fat, as well as saturated and unsaturated fatty acids, increased across the tertiles of the pattern, although this was only significant for saturated fatty acids (*p* = 0.0005) (Table 3).

The second dietary pattern explained 27.5% of the bile acid variance. This pattern was characterized by positive loadings for fried potatoes (0.4), margarine (0.35), high-fat plant-based milk products (0.27), potato chips (0.26), sauce (0.24), bread (0.21), and confectionery (0.20). DCA levels were lower across the tertiles of this dietary pattern, whereas levels of UDCA and taurine-conjugated bile acids were higher.

The remaining three dietary patterns explained only 5.1%, 2.7%, and 0.5% of bile acid variation and are not further discussed in this study.

## 4. Discussion

To the best of our knowledge, this is the first study investigating associations between serum bile acids in vegans and omnivores, and data on fecal bile acids in vegans are rare.

As expected, we observed higher dietary fiber intake and lower fecal bile acids in vegans compared to omnivores. Therefore, our results may confirm the association of lower fecal bile acids concentrations with high-fiber and low-fat diets [11,12,34], such as a vegan diet. In contrast, our results in serum in vegans compared to omnivores were rather unexpected. We found increased serum but decreased fecal bile acid concentrations in vegans as compared to omnivores. The increased amounts of serum bile acids in vegans were mostly due to increased levels of unconjugated primary bile acids, CA and CDCA, and glycine-conjugated bile acids. This might be explained by higher degrees of reabsorption of bile acid from terminal ileum (glycine-conjugated bile acid) and unconjugated primary bile acids (CA and CDCA) from colon, thus resulting in lower total fecal bile acid concentrations. These changes are nonetheless within physiological ranges, as hepatic bile acid synthesis, estimated by C4, was similar in both vegans and omnivores.

Although we observed significant differences in concentrations of bile acids in serum and feces between vegans and omnivores, the composition of bile acids differed only modestly between the two groups. Overall, the compositions of serum and fecal bile acids profiles in our study were in line with other observations of healthy populations with a high proportion of secondary bile acids in feces and conjugated bile acids in serum [40].

In healthy populations, glycine-conjugated bile acids have the highest proportion of total bile acids in serum [19,41,42,43], followed by the secondary bile acid DCA [41,42], in agreement with the observed serum bile acid profiles in both vegans and omnivores of our study. The conjugation of bile acids with either amino acid taurine or glycine may depend on diet. A diet high in animal products contains more taurine, meaning that taurine conjugation is more strongly linked to meat consumption [25], for example. Despite higher excreted taurine-conjugated bile acids in stools of omnivores, serum levels of taurine-conjugated bile acids did not differ between vegans and omnivores in our study. The conjugation of bile acids with glycine is associated with the intake of vegetables and carbohydrates due to the abundance of this amino acid in these food groups [20,25]. Thus, the higher intake of these food groups in vegans compared to omnivores in our study was in line with higher glycine-conjugated bile acids in serum in vegans and may confirm this hypothesis. Interestingly, fecal glycine-conjugated bile acid concentrations were lower in vegans than in omnivores, and this observation needs to be confirmed in further studies before drawing any conclusion.

In line with other cross-sectional studies in vegan populations [44,45,46], we observed a significant higher fiber intake in vegan participants compared to omnivorous participants. Due to their absorptive and viscous properties, fibers are suggested to “bind” bile acids by forming micelles with bile acids [47]. This interaction depends on the hydrophobic structure of bile acids, and, in an in vitro study, the more hydrophobic bile acids DCA and CDCA were absorbed more than CA [48]. The absorption rate may also depend on the type of fiber; thus, cellulose showed a higher binding capacity than lignin [10], and, among vegetables, kale showed a high binding of bile acids [49] in vitro. The micelles are suggested to inhibit the re-absorption of bile acids [50], and to enhance their excretion in stool [47]. However, our findings of higher fecal bile acid concentrations in omnivores and higher serum bile acid levels in vegans do not support this hypothesis and further human studies are needed to clarify the relevance of this mechanism in vivo.

We observed a significantly lower fat intake in vegans than in omnivores, which is line with other European cross-sectional studies investigating nutrient intake in vegan populations [44,45,46]. Fat intake alters bile acid concentrations [18]. In a previous study of healthy participants after three months of a high-fat/high-beef diet, fecal total and secondary bile acids increased compared to the levels observed in those participants following an ongoing mixed diet [14]. The effects of changes from animal-based to plant-based diets or vice versa on fecal bile acid concentrations were investigated in very small intervention studies. In a Dutch study, 12 participants were randomized to a vegetarian, vegan, or mixed diet group for 20 days, and total fecal bile acids and DCA decreased in the vegan and vegetarian groups compared to the mixed group [35]. In another recent cross-over study with 10 participants, total fecal bile acids and DCA increased after five days of consuming a diet with animal products compared to the plant-based diet [34]. After a two-week diet change from a high-fiber rural diet to a high-fat Westernized diet, fecal secondary bile acids increased in 20 native Africans [11]. In our cross-sectional study, concentrations of total fecal bile acids and secondary bile acids were lower in vegans than in omnivores and in agreement with the results of these short-term intervention studies.

Additionally, we applied RRR to derive dietary patterns explaining the variation in fecal bile acids. Another method for deriving dietary pattern is principal component analysis (PCA), which explains the maximum variation of food intakes. In contrast to PCA, the RRR approach allows the derivation of dietary patterns associated with biomarkers, thereby relating them to specific pathways or disease risk [32]. This is of interest in our study, since secondary bile acids are discussed with an increased risk of CRC, and, due to their role as signaling molecules, bile acids are part of metabolic diseases such as dyslipidemia [51] or type 2 diabetes [52].

A high score for the first pattern was characterized by a positive correlation with processed meat, fish, margarine, fried potatoes, and coffee, and a negative correlation with muesli. Although the consumption of fish, fried potatoes, and margarine was only observed in the 75th percentile of the participants, the derived dietary pattern explained 47.4% variance of bile acids and confirmed the applicability of the RRR as a statistical method.

As expected due to known [12,14,18] and discussed associations between diet and bile acid metabolism, dietary fat showed a positive, but fiber intake a negative correlation with this dietary pattern. The proportion of vegans decreased across the pattern score.

Secondary fecal bile acid concentrations increased across the tertiles of the first pattern. Accordingly, the concentrations of DCA and LCA reached higher levels in the pattern compared to observed concentrations in stools of omnivores. This might be explained by the increasing fat intake across the pattern and, thus, the increasing proportion of omnivores across the pattern. The variance of the derived pattern was mostly driven by glycine-conjugated bile acids, which is in line with the high proportion of glycine-conjugated bile acids in enterohepatic circulation.

This positive correlation of bile acids with the derived pattern confirms the impact of food products rich in fat and particularly of animal origin on elevated bile acid concentrations [11,12,17], as also described in previous studies. In particular, processed meat, which is high in cholesterol and saturated fatty acids [53], contributed to this dietary pattern. Increasing intake of saturated fatty acids in the derived pattern was in line with this observation. Moreover, our derived dietary pattern included fried potatoes, which were enriched with fat after processing, and their intake was correlated with higher bile acid concentration across the pattern. Yet, due to their fat content, the identification of processed meat, margarine, and fried potatoes as major food groups in this pattern was in line with a recent population based study, which observed a positive correlation between intakes of meat, processed meat, potatoes, and vegetables oils and fecal bile acid concentrations [54].

Nevertheless, the first derived pattern was inversely correlated with muesli intake, a food containing cereals and rich in fiber. This can be interpreted as in line with a study in Finnish women, which showed that, after adding rye bread (high in fiber) to the normal diet for two weeks, total fecal bile acids were lower compared to the baseline diet or after the consumption of low-fiber bread [55]. Moreover, the inverse association between fiber intake and the pattern score may support the hypothesis of the bile acid-binding capacities of fiber.

A few limitations merit consideration. A weakness of our study is that we did not estimate fecal bile acid excretion rates. Rather, bile acid concentrations were analyzed in aliquots of homogenized fresh stool samples instead of dry matter. Measurements of fecal bile acid excretion rates would have demanded complete stool collections over several days, which is notoriously infeasible in an ambulatory study setting outside dedicated clinical trial facilities. Rather, we presented significant differences in fecal bile acid concentrations between vegans and omnivores in aliquots of carefully homogenized morning bowel movements. We believe that this approach is justified as we did not observe significant differences in the total weights of these stool samples. Of note, this approach is not only in agreement with similar studies [35,56], but also supported by comparisons of bile acid concentrations between wet and dried fecal samples that did not find differences [40]. Moreover, our observations of increased fecal bile acids in omnivores compared to vegans are totally in line with literature [11,12,34,35] and confirm the associations of a plant-based diet and decreased bile acids in wet fecal mass.

Our aim was to identify a dietary pattern explaining the variation in fecal bile acid levels by implementing the RRR technique. Due to this narrow focus, our study did not address cross-linked information about microbiota composition or fecal short-chained fatty acids as bacterial metabolites out of fiber degradation. The investigation into these associations needs further research.

Three-day weighed food records were used to assess dietary intake. Although weighed food records are called the gold standard for assessing dietary intake, it is recognized in nutritional epidemiology that weighing dietary records are time-consuming for the participants and can be biased by influencing the eating habits during the protocol days or as a result of under-reporting [57]. Nevertheless, this instrument was chosen because the specific food items of a vegan diet were captured more reliably in weighing protocols than in standardized food frequency questionnaires. The small sample size of both groups and the high educational level of the participants may suggest that our study population lacked a good representation of the general population. However, the high degree of education could explain the low numbers of smokers and moderate levels of physical activity. Furthermore, the similar distribution of education and lifestyle characteristics in both groups may minimize the risk of confounding.

## 5. Conclusions

In our study, we observed a higher dietary fiber and lower fat intake, in line with lower fecal bile acid concentrations, in vegans compared to omnivores. We provide the first data on serum bile acids in vegans compared to omnivores.

Our findings could suggest that a vegan diet, which is low in fat and high in fiber intake, is related to lower fecal bile acid concentrations, and it may play a protective role in the development of CRC. Due to their role in metabolic regulation, bile acids may not only be relevant to the development of CRC, but also to metabolic diseases such as dyslipidemia [51] or type 2 diabetes [52].

In conclusion, despite an increasing trend toward veganism in Western countries, studies investigating associations between a vegan diet and metabolic changes are still scarce. If replicated in larger studies, the association between a vegan diet and fecal bile acids may be established as an important underlying mode of action to explain protective effects in the development of CRC and other metabolic diseases. 

## Figures and Tables

**Table 1:** Characteristics of the Risk and Benefits of a Vegan Diet study (RBVD) population.

	Vegan (*n* = 36)	Omnivore (*n* = 36)	*p*
Male (%)	50%	50%	
Age (years)	37.5 (32.5–44.0)	38.5 (32.0–46.0)	0.75
Body weight (kg)	70.1 ± 13.9	73.6 ± 10.3	0.24
BMI	22.9 ± 3.2	24.0 ± 2.1	0.08
Cholesterol (mg/dL)	165.9 ± 34.9	205.4 ± 41.6	<0.0001
Duration of vegan diet (years)	4.8 (3.1–8.7)	n.a.	
Education (*n* (%))			0.60
Low	0 (0.0)	1 (2.8)	
Intermediate	11 (30.6)	11 (30.6)	
High	25 (69.5)	24 (30.6)	
Physical activity (h/week)	2.8 (0.88–3.75)	2.3 (1.2–4.1)	0.69
Smoking behavior (*n* (%))			0.30
Never smoker	24 (66.7)	21 (58.3)	
Ex-smoker	8 (22.2)	6 (16.7)	
Smoker	4 (11.1)	9 (25)	
Stool			
Weight (mg)	102.6 (39.9–185.9)	97.3 (47.4–157.6)	0.79
Processing time (h:min)	2:48 (1:49–3:45)	2:32 (1:34–3:38)	0.47

Data are reported as means (± SD) for normally distributed variables, as medians (quartile (Q)1–Q3) for variables not normally distributed, or as percentage (%). Low education: no degree; intermediate education: vocational school, technical college; high education: university, university of applied sciences. Statistical tests were carried out using the *t*-test for normally distributed variables, Mann–Whitney U test for variables not normally distributed, and chi^2^ for categorical variables. BMI = body mass index; n.a. = not applicable.

**Table 2:** Concentrations of fecal bile acids across first pattern score.

	Tertiles of Dietary Pattern Score			
Bile Acids (nmol/g)	1 (75% Vegans)	2 (50% Vegans)	3 (25% Vegans)	*p*-Value for Trend
DCA	74.23 (3.22–195.81)	586.21 (283.57–1041.85)	1327.30 (644.51–3037.96)	<0.0001
LCA	64.20 (13.99–93.47)	198.93 (120.73–327.30)	417.49 (305.10–1070.67)	<0.0001
UDCA	0.01 (0.00–0.16)	0.16 (0.00–0.64)	1.07 (0.00–16.91)	0.02
Total glycine-con.	0.73 (0.32–1.52)	3.45 (2.43–5.95)	9.06 (5.76–18.81)	<0.0001
Total taurine-con.	0.26 (0.05–0.50)	1.05 (0.49–2.00)	3.25 (1.71–8.65)	<0.0001

Concentrations of fecal bile acids are presented as medians (Q1–Q3). DCA = deoxycholic acid, LCA = lithocholic acid, UDCA = ursodeoxycholic acid, con. = conjugated.

**Table 3:** Intake of macronutrients according to tertiles of the first dietary pattern.

	Tertiles of Dietary Pattern Score			
Macronutrients (g/day)	1 (75% Vegans)	2 (50% Vegans)	3 (25% Vegans)	*p*-Value for Trend
Fiber	37 (25–55)	32 (27–50)	24 (17–34)	0.01
Protein	75 (53–122)	90 (68–107)	78 (69–103)	0.43
Fat	92 (64–109)	100 (83–124)	98 (85–152)	0.05
Carbohydrates	238 (203–310)	236 (205–297)	270 (211–356)	0.37
Sucrose	46 (34–61)	53 (37–68)	60 (41–81)	0.19
Unsaturated fatty acids	34 (23–40)	34 (28–43)	33 (27–49)	0.25
Saturated fatty acids	19 (10–33)	36 (16–44)	34 (26–52)	0.0005

Data are presented as medians (Q1–Q3).

## Supplementary Materials

The following are available online at https://www.mdpi.com/2072-6643/12/1/47/s1: Table S1: Yielding factors for prepared food items; Table S2: Definition of food groups; Table S3: Intake of food groups of RBVD population; Table S4: Factor loadings of all 53 food groups in first dietary pattern score; Figure S1: Study flow chart.

## References

[1] Mensink G., Lage Barbosa C., Brettschneider A. (2016). Verbreitung der vegetarischen Ernährungsweise in Deutschland. J. Health Monit..

[2] Janssen M., Busch C., Rodiger M., Hamm U. (2016). Motives of consumers following a vegan diet and their attitudes towards animal agriculture. Appetite.

[3] Tonstad S., Butler T., Yan R., Fraser G.E. (2009). Type of vegetarian diet, body weight, and prevalence of type 2 diabetes. Diabetes Care.

[4] Le L.T., Sabate J. (2014). Beyond meatless, the health effects of vegan diets: Findings from the Adventist cohorts. Nutrients.

[5] Appleby P.N., Key T.J. (2016). The long-term health of vegetarians and vegans. Proc. Nutr. Soc..

[6] Benatar J.R., Stewart R.A.H. (2018). Cardiometabolic risk factors in vegans; A meta-analysis of observational studies. PLoS ONE.

[7] Dinu M., Abbate R., Gensini G.F., Casini A., Sofi F. (2017). Vegetarian, vegan diets and multiple health outcomes: A systematic review with meta-analysis of observational studies. Crit. Rev. Food Sci. Nutr..

[8] World Cancer Research Fund International Colorectal Cancer Statistics. https://www.wcrf.org/dietandcancer/cancer-trends/colorectal-cancer-statistics.

[9] Thanikachalam K., Khan G. (2019). Colorectal cancer and nutrition. Nutrients.

[10] Singh J., Metrani R., Shivanagoudra S.R., Jayaprakasha G.K., Patil B.S. (2019). Review on bile acids: Effects of the gut microbiome, interactions with dietary fiber, and alterations in the bioaccessibility of bioactive compounds. J. Agric. Food Chem..

[11] O’Keefe S.J., Li J.V., Lahti L., Ou J., Carbonero F., Mohammed K., Posma J.M., Kinross J., Wahl E., Ruder E. (2015). Fat, fibre and cancer risk in African Americans and rural Africans. Nat. Commun..

[12] Ou J., Carbonero F., Zoetendal E.G., DeLany J.P., Wang M., Newton K., Gaskins H.R., O’Keefe S.J.D. (2013). Diet, microbiota, and microbial metabolites in colon cancer risk in rural Africans and African Americans. Am. J. Clin. Nutr..

[13] Thorning T.K., Raziani F., Bendsen N.T., Astrup A., Tholstrup T., Raben A. (2015). Diets with high-fat cheese, high-fat meat, or carbohydrate on cardiovascular risk markers in overweight postmenopausal women: A randomized crossover trial. Am. J. Clin. Nutr..

[14] Reddy B.S. (1981). Diet and excretion of bile acids. Cancer Res..

[15] Chiang J.Y. (2017). Recent advances in understanding bile acid homeostasis. F1000Research.

[16] Hahn C., Reichel C., von Bergmann K. (1995). Serum concentration of 7 alpha-hydroxycholesterol as an indicator of bile acid synthesis in humans. J. Lipid Res..

[17] Ridlon J.M., Wolf P.G., Gaskins H.R. (2016). Taurocholic acid metabolism by gut microbes and colon cancer. Gut Microbes.

[18] Wahlstrom A., Sayin S.I., Marschall H.U., Backhed F. (2016). Intestinal crosstalk between bile acids and microbiota and its impact on host metabolism. Cell Metab..

[19] Fiamoncini J., Yiorkas A.M., Gedrich K., Rundle M., Alsters S.I., Roeselers G., van den Broek T.J., Clavel T., Lagkouvardos I., Wopereis S. (2017). Determinants of postprandial plasma bile acid kinetics in human volunteers. Am. J. Physiol. Gastrointest. Liver Physiol..

[20] De Aguiar Vallim T.Q., Tarling E.J., Edwards P.A. (2013). Pleiotropic roles of bile acids in metabolism. Cell Metab..

[21] Di Ciaula A., Garruti G., Lunardi Baccetto R., Molina-Molina E., Bonfrate L., Wang D.Q., Portincasa P. (2017). Bile acid physiology. Ann. Hepatol..

[22] Ajouz H., Mukherji D., Shamseddine A. (2014). Secondary bile acids: An underrecognized cause of colon cancer. World J. Surg. Oncol..

[23] Bernstein H., Bernstein C., Payne C.M., Dvorak K. (2009). Bile acids as endogenous etiologic agents in gastrointestinal cancer. World J. Gastroenterol..

[24] Ridlon J.M., Kang D.J., Hylemon P.B., Bajaj J.S. (2014). Bile acids and the gut microbiome. Curr. Opin. Gastroenterol..

[25] Ridlon J.M., Kang D.J., Hylemon P.B. (2006). Bile salt biotransformations by human intestinal bacteria. J. Lipid Res..

[26] O’Keefe S.J. (2016). Diet, microorganisms and their metabolites, and colon cancer. Nat. Rev. Gastroenterol. Hepatol..

[27] Molinaro A., Wahlstrom A., Marschall H.U. (2018). Role of bile acids in metabolic control. Trends Endocrinol. Metab..

[28] Shapiro H., Kolodziejczyk A.A., Halstuch D., Elinav E. (2018). Bile acids in glucose metabolism in health and disease. J. Exp. Med..

[29] Hu F.B. (2002). Dietary pattern analysis: A new direction in nutritional epidemiology. Curr. Opin. Lipidol..

[30] Frank L.K., Jannasch F., Kroger J., Bedu-Addo G., Mockenhaupt F.P., Schulze M.B., Danquah I. (2015). A dietary pattern derived by reduced rank regression is associated with type 2 diabetes in an urban Ghanaian population. Nutrients.

[31] Schulz M., Hoffmann K., Weikert C., Nothlings U., Schulze M.B., Boeing H. (2008). Identification of a dietary pattern characterized by high-fat food choices associated with increased risk of breast cancer: The European Prospective Investigation into Cancer and Nutrition (EPIC)-Potsdam study. Br. J. Nutr..

[32] Hoffmann K., Schulze M.B., Schienkiewitz A., Nothlings U., Boeing H. (2004). Application of a new statistical method to derive dietary patterns in nutritional epidemiology. Am. J. Epidemiol..

[33] Weikert C., Schulze M.B. (2016). Evaluating dietary patterns: The role of reduced rank regression. Curr. Opin. Clin. Nutr. Metab. Care.

[34] David L.A., Maurice C.F., Carmody R.N., Gootenberg D.B., Button J.E., Wolfe B.E., Ling A.V., Devlin A.S., Varma Y., Fischbach M.A. (2014). Diet rapidly and reproducibly alters the human gut microbiome. Nature.

[35] Van Faassen A., Bol J., Van Dokkum W., Pikaar N.A., Ockhuizen T., Hermus R.J.J. (1987). Bile acids, neutral steroids, and bacteria in feces as affected by a mixed, a lacto-ovovegetarian, and a vegan diet. Am. J. Clin. Nutr..

[36] Bognar A. (2002). Tables on Weight Yield of Food and Retention Factors of Food Constituents for the Calculation of Nutrient Composition of Cooked Foods (Dishes).

[37] Schulze M.B., Hoffmann K., Kroke A., Boeing H. (2001). Dietary patterns and their association with food and nutrient intake in the European Prospective Investigation into Cancer and Nutrition (EPIC)-Potsdam study. Br. J. Nutr..

[38] Kroger J., Ferrari P., Jenab M., Bamia C., Touvier M., Bueno-de-Mesquita H.B., Fahey M.T., Benetou V., Schulz M., Wirfalt E. (2009). Specific food group combinations explaining the variation in intakes of nutrients and other important food components in the European prospective investigation into cancer and nutrition: An application of the reduced rank regression method. Eur. J. Clin. Nutr..

[39] Weikert C., Hoffmann K., Dierkes J., Zyriax B.C., Klipstein-Grobusch K., Schulze M.B., Jung R., Windler E., Boeing H. (2005). A homocysteine metabolism-related dietary pattern and the risk of coronary heart disease in two independent German study populations. J. Nutr..

[40] Chen W., Wei Y., Xiong A., Li Y., Guan H., Wang Q., Miao Q., Bian Z., Xiao X., Lian M. (2019). Comprehensive analysis of serum and fecal bile acid profiles and interaction with gut microbiota in primary biliary cholangitis. Clin. Rev. Allergy Immunol..

[41] Luo L., Aubrecht J., Li D., Warner R.L., Johnson K.J., Kenny J., Colangelo J.L. (2018). Assessment of serum bile acid profiles as biomarkers of liver injury and liver disease in humans. PLoS ONE.

[42] Wewalka M., Patti M.E., Barbato C., Houten S.M., Goldfine A.B. (2014). Fasting serum taurine-conjugated bile acids are elevated in type 2 diabetes and do not change with intensification of insulin. J. Clin. Endocrinol. Metab..

[43] Xie G., Wang Y., Wang X., Zhao A., Chen T., Ni Y., Wong L., Zhang H., Zhang J., Liu C. (2015). Profiling of serum bile acids in a healthy Chinese population using UPLC-MS/MS. J. Proteome Res..

[44] Elorinne A.L., Alfthan G., Erlund I., Kivimaki H., Paju A., Salminen I., Turpeinen U., Voutilainen S., Laakso J. (2016). Food and nutrient intake and nutritional status of Finnish vegans and non-vegetarians. PLoS ONE.

[45] Kristensen N.B., Madsen M.L., Hansen T.H., Allin K.H., Hoppe C., Fagt S., Lausten M.S., Gobel R.J., Vestergaard H., Hansen T. (2015). Intake of macro- and micronutrients in Danish vegans. Nutr. J..

[46] Schupbach R., Wegmuller R., Berguerand C., Bui M., Herter-Aeberli I. (2017). Micronutrient status and intake in omnivores, vegetarians and vegans in Switzerland. Eur. J. Nutr..

[47] Gunness P., Gidley M.J. (2010). Mechanisms underlying the cholesterol-lowering properties of soluble dietary fibre polysaccharides. Food Funct..

[48] Naumann S., Schweiggert-Weisz U., Eglmeier J., Haller D., Eisner P. (2019). In Vitro interactions of dietary fibre enriched food ingredients with primary and secondary bile acids. Nutrients.

[49] Yang I.F., Jayaprakasha G.K., Patil B.S. (2017). In vitro bile acid binding capacities of red leaf lettuce and cruciferous vegetables. J. Agric. Food Chem..

[50] Naumann S., Schweiggert-Weisz U., Bader-Mittermaier S., Haller D., Eisner P. (2018). Differentiation of adsorptive and viscous effects of dietary fibres on bile acid release by means of in vitro digestion and dialysis. Int. J. Mol. Sci..

[51] Breuninger T.A., Wawro N., Meisinger C., Artati A., Adamski J., Peters A., Grallert H., Linseisen J. (2019). Associations between fecal bile acids, neutral sterols, and serum lipids in the KORA FF4 study. Atherosclerosis.

[52] Prawitt J., Caron S., Staels B. (2011). Bile acid metabolism and the pathogenesis of type 2 diabetes. Curr. Diabetes Rep..

[53] Rohrmann S., Linseisen J. (2016). Processed meat: The real villain?. Proc. Nutr. Soc..

[54] Mitry P., Wawro N., Sharma S., Kriebel J., Artati A., Adamski J., Heier M., Meisinger C., Thorand B., Grallert H. (2019). Associations between usual food intake and faecal sterols and bile acids: Results from the Cooperative Health Research in the Augsburg Region (KORA FF4) study. Br. J. Nutr..

[55] Korpela J.T., Korpela R., Adlercreutz H. (1992). Fecal bile acid metabolic pattern after administration of different types of bread. Gastroenterology.

[56] Vaughn B.P., Kaiser T., Staley C., Hamilton M.J., Reich J., Graiziger C., Singroy S., Kabage A.J., Sadowsky M.J., Khoruts A. (2019). A pilot study of fecal bile acid and microbiota profiles in inflammatory bowel disease and primary sclerosing cholangitis. Clin. Exp. Gastroenterol..

[57] Thompson F.E., Subar A.F., Coulston A.M., Boushey C.J., Ferruzzi M.G., Delahanty L.M. (2017). Chapter 1—Dietary assessment methodology. Nutrition in the Prevention and Treatment of Disease.

