# Supplementary material for: Associations between Dietary Patterns and Bile Acids—Results from a Cross-Sectional Study in Vegans and Omnivores
Source: Nutrients. 2019 Dec 23;12(1):47. doi: 10.3390/nu12010047 (PMC7019893; doi:10.3390/nu12010047)
Supplement: Supplementary file 1 [file nutrients-12-00047-s001.pdf]

Associations between dietary patterns and bile acids – results from a cross-sectional study in vegans and omnivores

Iris Trefflich[1,4]*, Hanns-Ulrich Marschall[2], Romina di Giuseppe[3], Marcus Ståhlman[2], Andreas Michalsen[4], Alfonso Lampen[1], Klaus Abraham[1] and Cornelia Weikert[1,4]

[1]German Federal Institute for Risk Assessment, Department of Food Safety, Berlin, Germany;
[2]University of Gothenburg, Department of Molecular and Clinical Medicine, Sweden
[3] Romina di Giuseppe, Institute for Epidemiology, University Kiel, Germany
[4]Institute for Social Medicine, Epidemiology and Health Economics, Charité-University Medicine, Berlin
* Correspondence: iris.trefflich@bfr.bund.de

Supplemental material

Supplemental Figure S1: Study flow chart

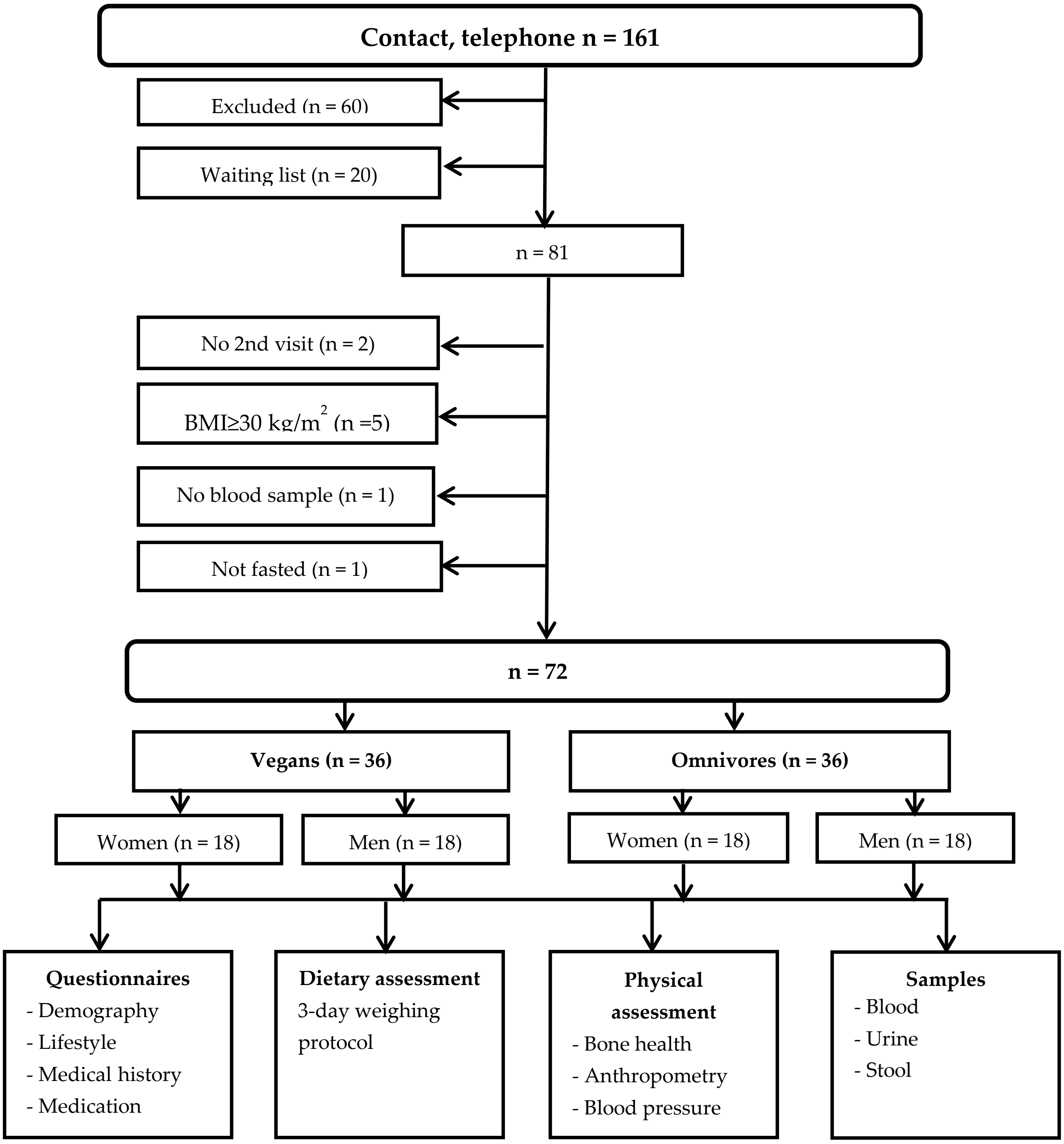

Supplemental Table S1: Yielding factors for prepared foot items

**Food items**	**Factor**
Eggs	0,91
Meat / Calf	0,86
Meat / beef	0,81
Meat / pork, cooked	0,78
Meat / pork, fried	0,74
Meat / mix, Burger	0,93
Meat / lamb	0,63
Meat / offal	0,97
Meat / sausages	0,78
Meat / poultry, cooked	0,41
Meat / poultry, fried	0,84
Fish, cooked	0,5
Fish, fried	0,72
Soups	12,05
Soups, Meat	11,57
Stew	13,72
Bouillon	39,3
Sauces	6,6
Stew, legumes	3,04
Stew vegetable/meat	0,95
Vegetables, root	0,92
Vegetables, leaves	1
Vegetables, fruit (sweet pepper, cauliflower, pumpkin)	0,98
Potatoes, cooked	0,97
Legumes, dry	3,1
Legumes	1,04
Potatoes, fried	0,68
Cereals / rice, dry	3,68
Pasta, dry	2,51
Pasta, fresh	1,02
Bread, cake, pizza	0,87

Yielding factors are based on Bognar et al. (2002). For calculation the actual consumption amount of prepared dishes, quantities of food items are in recipes were multiplied with respectively factor.

Supplemental Table S2: Definition of food groups

Food group	Description
Cooked vegetables	Tomatoes, tomato sauce, sweet pepper, eggplant, spinach, carrots, asparagus, peas, carrot vegetable mix, leek, celery (all cooked)
Cabbage family	Broccoli, cauliflower, red and white cabbage, kohlrabi (all cooked)
Legumes	Green peas, green beans, pea, bean, lentil stew
Cooked potatoes	Salted potatoes, jacket potatoes, mashed potatoes, potato salad, dumplings
Mushrooms	Fresh mushrooms, mushroom dishes
Sauce	Ketchup, brown and white sauce, salad dressing, sauce for vegetables
Poultry	Fried, grilled or roasted chicken or turkey
Meat except fish and poultry	Pork, beef, hamburger, minced meat, liver, lamb, roast hare
Animal fat except butter	Animal fat used for food preparation
Dessert*	Pudding, sweet soufflé
Cake, cookies*	Cake, tart, cookies
Confectionery, ice cream*	Chocolate, candy bars, pralines, sugar, ice-cream, cereal bars
Jam, honey, chocolate spread*	Jam, honey, chocolate spread, peanut butter, cashew butter, almond butter
Canned fruit	Canned fruit
Fruit juice	Citrus, apple, orange, grapefruit, grape, cherry, pineapple juice, multi-vitamin drinks, vegetable juices
Tea	Black tea, green tea, fruit and herbal teas
Muesli	Whole-grain breakfast cereal, muesli
Cornflakes	Cornflakes, other refined grain-based breakfast cereal
Pasta*, rice	Cooked pasta, cooked rice, other cooked cereals (millet, bulgur, quinoa)
Pizza*	Pizza, quiche
Vegetarian dishes	Vegetarian dishes
Garlic	Raw, fried or cooked garlic
Whole meal bread	Whole meal bread, dark and whole meal rolls
Other bread	Rye bread, wheat bread, mixed bread, pale rolls, crispbread, croissants
Olive oil	Olive oil used for food preparation
Fruits	
Fresh fruits	All fresh fruits including avocado
Dried fruits	Raisins, dates, figs, acai berry and all other dried fruits
Raw vegetables	Cucumber, carrot, sprouts, paprika, tomato, onion, radish, olives
Other vegetables oils and fat	Vegetable fat used for food preparation (frying, dressing etc.) incl. coconut oil
Water	Tap water, mineral water
Fish	Fish, canned fish, smoked fish
Nuts	Nuts, Chia seed, pumpkin, sunflower, linseed
Chips, salt sticks	Chips, salt sticks, cracker
Fried potatoes	French fries, potato fritters, fried potatoes
Beer	Beer
Spirits	Spirits
Wine	Wine, fruit wine, champagne
Other alcoholic beverages	Dessert wine, liqueur, aperitif
Eggs	Boiled eggs, fried eggs, omelet
Coffee	Coffee
Soup	Vegetable or potato stew, vegetable soup, meat or fish soup, broth, thickened soup

Food group	Description
Processed meat	Salami, cold-cut sausage, ham, fried sausage
Low-fat dairy products	Milk or yoghurt (<=1,5 % fat), soured milk, low-fat curd cheese
High-fat dairy products	Other milk or yoghurt, curd cheese, cream
Low-fat cheese	Low-fat cheese <=45%
High-fat cheese	Other cheese
Butter	Butter as spread and for food preparation
Margarine	Margarine as spread and for food preparation
*Plant-based milk***	Plant based milks
Soy milk	Soy milk
Cereal based milk	Wheat, oat, spelt milk
Nut based milk	Almond, coconut, hazelnut milk
Plant-based milk products (low fat)	plant based cheese, yoghurt, cheese curd
Plant-based milk products (high fat)	plant based cream
Meat alternatives	Tofu, seitan, tempeh, processed products e.g. sausages, burger
Vegetarian spread	vegetables based spreads, hummus
Soft drink and other non-alcoholic drinks	Soft drink and other non-alcoholic drinks

Food groups used for analysis of 3-weighing protocols. *Food groups combined vegan and omnivorous food items, but were captured separately for vegans and omnivores. **Plant based milk included soy milk, nut-based milk and cereal based milk. Food groups in italic letters were added new for this study.

Supplemental Table S3. Intake of food groups of RBVD population

Food group	Vegan	Omnivore	p
Cooked vegetable	115.83 (56.33-207.17)	71.67 (15.67-112.67)	0.008
Cabbage family	21.17 (0.00-48.17)	0.00 (0.00-21.50)	0.03
Legumes	58.50 (0.00-105.33)	0.00 (0.00-9.17)	<.0001
Cooked potatoes	7.50 (0.00-81.84)	0.00 (0.00-76.50)	0.48
Mushrooms	0.00 (0.00-15.67)	0.00 0.00-0.00	0.16
Sauce	3.50 (0.00-12.33)	5.83 (0.00-30.50)	0.33
Poultry	0.00 (0.00-0.00)	14.67 (0.00-61.17)	<.0001
Meat except fish and poultry	0.00 (0.00-0.00)	18.33 (0.00-85.00)	<.0001
Animal fat except butter	0.00 (0.00-0.00)	0.00 (0.00-0.00)	0.15
Dessert*	0.00 (0.00-0.00)	0.00 (0.00-0.00)	0.67
Cake, cookies*	0.00 (0.00-26.84)	19.33 (0.00-63.83)	0.12
Confectionary, ice cream*	29.67 (7.50-74.67)	29.33 (9.00-55.33)	0.90
Jam, honey, chocolate spread*	0.00 (0.00-11.67)	2.17 (0.00-8.83)	0.63
Canned fruit	0.00 (0.00-0.00)	0.00 (0.00-0.00)	0.98
Fruit juice	18.83 (0.00-162.17)	78.34 (0.00-205.17)	0.83
Tea	266.50 (0.00-903.33)	0.00 (0.00-300.00)	0.04
Muesli	27.67 (0.00-56.50)	0.00 (0.00-28.17)	0.11
Cornflakes	0.00 (0.00-0.00)	0.00 (0.00-0.00)	0.30
Pasta*, rice	107.00 (48.33-203.00)	56.17 (0.00-126.00)	0.05
Pizza*	0.00 (0.00-0.00)	0.00 (0.00-0.00)	0.88
Vegetarian dishes	0.00 (0.00-0.00)	0.00 (0.00-0.00)	0.25
Garlic	0.00 (0.00-1.67)	0.00 (0.00-0.33)	0.07
Whole grain bread	4.00 (0.00-39.17)	0.00 (0.00-19.00)	0.12
Other bread	44.50 (9.00-106.67)	96.33 (58.17-149.33)	<..01
Olive oil	2.17 (0.00-7.84)	4.50 (0.00-10.17)	0.54
Fresh fruit	185.50 (94.67-344.83)	152.33 (62.17-215.17)	0.14
Raw vegetables	145.67 (77.33-327.67)	76.67 (43.67-148.67)	0.01
Vegetables oils and fat	12.67 (4.83-27.67)	1.50 (0.00-8.00)	<.0001
Water	1558.83 (1006.50-2089.00)	1128.50 (595.501866.33)	0.05
Fish	0.00 (0.00-0.00)	0.00 (0.00-38.83)	0.005
Nuts and Seeds	18.50 (2.69-41.17)	1.17 (0.00-18.00)	0.01
Chips, salt sticks	0.00 (0.00-0.00)	0.00 (0.00-0.00)	1.00
Fried potatoes	0.00 (0.00-5.50)	0.00 (0.00-0.00)	0.54
Beer	0.00 (0.00-0.00)	0.00 (0.00-25.00)	0.62
Spirits	0.00 (0.00-0.00	0.00 (0.00-0.00)	0.15
Wine	0.00 (0.00-0.00	0.00 (0.00-41.34)	0.01
Other alcoholic beverages	0.00 (0.00-0.00	0.00 (0.00-0.00)	0.32
Eggs	0.00 (0.00-0.00	17.17 (0.00-39.50)	<.0001
Coffee	81.83 (0.00-285.33)	172.00 (49.50-344.33)	0.06
Soup	0.00 (0.00-2.67)	0.00 (0.00-32.33)	0.54
Processed meat	0.00 (0.00-0.00)	31.50 (3.17-76.83)	<.0001
Low-dairy products	0.00 (0.00-0.00)	238.67 (121.33-427.00)	<.0001
High-dairy products	0.00 (0.00-0.00)	0.00 (0.00-53.33)	<.0001
Low fat cheese	0.00 (0.00-0.00)	13.17 (0.00-33.50)	<.0001
High fat cheese	0.00 (0.00-0.00)	4.00 (0.00-20.00)	<.0001
Butter	0.00 (0.00-0.00)	14.67 (2.17-21.50)	<.0001
Margarine	0.00 (0.00-2.50)	0.00 (0.00-0.00)	0.20
Plant-based milk	184.50 (76.00-304.67)	0.00 (0.00-0.00)	<.0001
Plant-based milk products low fat	29.50 (0.00-74.84)	0.00 (0.00-0.00)	<.0001
Plant-based milk products high fat	0.00 (0.00-13.84)	0.00 (0.00-0.00)	<.001
Meat alternatives	64.00 (11.34-120.33)	0.00 (0.00-0.00)	<.0001
Savory spread plant-based	10.67 (0.0030.00)	0.00 (0.00-0.00)	<.0001
Soft drinks	0.00 (0.00-83.83)	0.00 (0.00-161.84)	0.84

Median (Q1-Q3) intake of food groups of RBVD population (g/d), based on weighing protocols. Statistical test: Mann Whitney-U Test. *Vegan alternative for this food groups was considered in results, e.g. egg-free pasta, vegan ice-cream or cakes.

Supplemental S4: Factor loadings of all 53 food groups in first dietary pattern score

Food group	Factor loading
Processed meat	0,26*
Fried potatoes	0,25*
Bread, other	0,25*
Margarine	0,24*
Fish	0,23*
Coffee	0,22*
Cheese, low fat	0,20
Sauce	0,19
Fruit juice	0,19
Dairy, low fat	0,18
Butter	0,15
Confectionary	0,15
Poultry	0,15
Dairy, high fat	0,14
Beer	0,12
Eggs	0,12
Animal fat	0,12
Meat	0,11
Wine	0,10
Cheese, high fat	0,10
Chips, salt sticks	0,10
Spirits	0,10
Soup	0,08
Soft drinks	0,08
Cake, Cookies	0,07
Vegetarian dishes	0,07
Cornflakes	0,07
Cooked potatoes	0,02
Garlic	0,02
Olive oil	-0,02
Plant based milk products, low fat	-0,03
Pizza	-0,03
Alcoholic beverages	-0,03
Dessert	-0,05
Jam, honey	-0,05
Whole meal bread	-0,06
Canned fruit	-0,06
Cooked vegetables	-0,08
Meat alternatives	-0,09
Raw vegetables	-0,10
Fresh fruit	-0,10
Plant based milk products, high fat	-0,11
Mushrooms	-0,11
Vegetable oil / fat	-0,11
Cabbage family	-0,11
Pasta, rice	-0,12
Water	-0,12
Legumes	-0,13
Tea	-0,13
Plant based milk	-0,15
Savory spreads	-0,17
Nuts, seeds	-0,18
Muesli	-0,23*

Factor loadings are correlation between food groups and the dietary pattern score. Food groups with factor loadings > 0.2 contributed to the simplified dietary pattern (marked with *)
